# Lung Cancer Driver Mutations in Middle Eastern Americans: Associations with Smoking and Comparative Analysis with Middle Eastern Populations

**DOI:** 10.1055/s-0046-1819575

**Published:** 2026-03-24

**Authors:** Amanda Henderson, Ann-Marie Zheng, Susan Daraiseh, Said Khayyata

**Affiliations:** 1 Oakland University William Beaumont School of Medicine, Michigan; 2 Department of Pathology and Laboratory Medicine, Corewell Health William Beaumont University Hospital, Royal Oak, Michigan, United States

**Keywords:** lung carcinoma, Middle Eastern, next-generation sequencing

## Abstract

**Background:**

Middle Eastern Americans represent a growing and understudied population in cancer genomics. While lung cancer driver mutations are well characterized in many populations, data remain limited for individuals of Middle Eastern ancestry living in the United States. Smoking histories were observed, but available evidence in this population is sparse.

**Methods:**

We conducted a retrospective, descriptive analysis of lung carcinoma specimens from 31 Middle Eastern American patients who underwent next-generation sequencing (NGS). Mutations in KRAS, EGFR, ALK, BRAF, and MET were identified using targeted NGS panels. Smoking exposure and duration of U.S. residence were summarized descriptively. Mutation frequencies were compared with published datasets from Middle Eastern populations, including My Cancer Genome and the American University of Beirut Medical Center. NGS using the formalin-fixed paraffin-embedded block was performed using the TruSight Oncology 500 Kit or the TruSight Tumor 15 Kit (Illumina, San Diego, California, United States). Data analysis was performed by aligning to human genome assembly GRCh37 (hg19). A custom variant filter was set up to include only nonsynonymous variants with coding consequences and read depth greater than 100X. Variants classified as IA or IB in ALK, BRAF, EGFR, KRAS, and MET were compared in this study. Benign variants, based on the ClinVar database, were excluded. Given the limited sample size, analyses were descriptive in nature. Continuous variables are presented as means, and categorical variables as counts and percentages. No inferential statistical testing was performed due to insufficient statistical power to reliably detect associations between smoking history and mutation status. The study is intended to be hypothesis-generating

**Results:**

KRAS mutations were the most frequently observed (38.7%), followed by EGFR (19.4%) and ALK (9.7%). EGFR mutations occurred predominantly in individuals with minimal smoking exposure, whereas KRAS mutations were more common among individuals with higher pack-year histories. BRAF and MET mutations were rare. Due to limited sample size, no inferential statistical testing was performed, and findings should be interpreted as exploratory.

**Conclusion:**

This descriptive study characterizes lung cancer driver mutations in Middle Eastern Americans and identifies patterns consistent with known smoking-associated and non-smoking-associated mutation profiles. While limited by sample size, the findings emphasize the importance of including Middle Eastern Americans in cancer genomic research and larger, statistically powered studies incorporating additional smoking history and ancestry data are needed to clarify these associations and their clinical implications. KRAS mutations were the most common mutations in our study, occurring in 38.71% of cases, with an average of 10.42 years of residence in the United States and 27.3 pack-years of smoking exposure. EGFR mutations are found in 19.35% of cases, with relatively low smoking exposure (1.25 pack-year). Both MET and BRAF mutations are rare, each accounting for 3.23% of the cases. BRAF mutations were detected in patients with the highest smoking exposure (70 pack-years); however, it should be noted that this value is elevated due to only having one positive case in this study. ALK mutations account for 9.68% of cases. On average, the study population spent 8.58 years of residency in the United States and 22.67 pack-years of smoking exposure, demonstrating a range of smoking histories and mutation frequencies. These findings were compared with data from My Cancer Genome, the American University of Beirut Medical Center, and specific EGFR mutation rates in the Middle Eastern population. Understanding these dynamics is essential for developing targeted public health strategies that address the unique challenges faced by this population, balancing their cultural heritage with the realities of modern American life. Further analysis and wider scope studies are necessary to explore the implications of these findings on health outcomes.

## Introduction

The Middle Eastern American population represents a growing and diverse group within the United States, with individuals originating from a variety of Middle Eastern countries. 1 Over the past several decades, there has been increasing interest in understanding the health disparities that affect Middle Eastern Americans, with a particular focus on how cultural, smoking history, and environmental factors shape their health outcomes. 2 3 One such critical area is cancer, specifically lung carcinoma, which remains one of the leading causes of cancer-related deaths in both the United States and worldwide. 4

Genetic mutations are well-established as key drivers of cancer, including lung carcinoma. Mutations in specific genes such as KRAS, EGFR, BRAF, MET, and ALK are known to contribute to tumorigenesis in lung cancer. 5 However, the impact of genetic mutations in lung carcinoma among Middle Eastern Americans has not been widely studied. Moreover, while genetic predispositions related to Middle Eastern heritage may play a role, it is essential to consider the influence of smoking history, particularly those associated with Westernization and migration.

Studies have shown that smoking history and other factors such as diet and physical activity can significantly affect the expression of genetic mutations. 6 7 Smoking, for example, has long been recognized as a primary risk factor for lung cancer and is known to induce specific genetic mutations, particularly in genes like KRAS and BRAF. However, Middle Eastern American populations may experience unique challenges and exposures due to cultural and environmental shifts after migration.

This study aims to provide a comprehensive analysis of the genetic mutations associated with lung carcinoma in Middle Eastern Americans, comparing the mutation profiles of this group to those observed in populations from their countries of origin in the Middle East. We hypothesize that lifestyle factors, such as smoking, may be influencing the genetic landscape of lung cancer in Middle Eastern Americans, and that these mutations may differ from those seen in Middle Eastern populations due to environmental changes following migration. By investigating these factors, we hope to contribute to the growing body of knowledge surrounding cancer in Middle Eastern American populations and provide insights for targeted public health strategies.

## Methods

A retrospective comparative analysis was conducted using tissue samples from *Middle Eastern* Americans diagnosed with lung carcinoma. Next-generation sequencing (NGS) was employed to identify genetic mutations, with a particular focus on nonsynonymous variants that are linked to cancer pathogenesis. For this analysis, formalin-fixed paraffin-embedded tissue blocks were processed using the TruSight Oncology 500 Kit or the TruSight Tumor 15 Kit (Illumina, San Diego, California, United States). Sequence data were aligned to the human genome assembly GRCh37 (hg19) using standard bioinformatics pipelines. A custom variant filter was implemented to include only nonsynonymous variants with coding consequences and a read depth greater than 100X. Variants classified as IA or IB in genes such as ALK, BRAF, EGFR, KRAS, and MET were specifically targeted for analysis. Variants classified as Tier IA or IB were defined according to established clinical significance criteria, representing variants with strong or potential clinical relevance, respectively, based on existing therapeutic, prognostic, or diagnostic evidence. 8 Benign variants were excluded using data from the ClinVar database. Results were then compared with mutation frequencies reported in Middle Eastern populations, including data from My Cancer Genome and the American University of Beirut Medical Center.

## Results

Our study of Middle Eastern American lung cancer patients observed that 76.9% of female lung cancer patients (10 out of 13) were smokers. On the other hand, there was a 61.8% smoking prevalence among male patients in our Middle Eastern American cohort (21 out of 34).

The analysis revealed significant differences in the mutation profiles of Middle Eastern Americans compared with Middle Eastern populations (see Tables 1 and 2 ):

- KRAS mutations were the most frequent, occurring in 38.71% of cases. These mutations were most commonly associated with an average of 10.42 years of residence in the United States. and 27.3 pack-years of smoking exposure.
- EGFR mutations were found in 19.35% of cases, with relatively low smoking exposure (average 1.25 pack-years). 9 EGFR mutations were observed predominantly in individuals with minimal smoking exposure, a pattern consistent with previously reported mutation distributions in nonsmoking populations.
- MET and BRAF mutations were rare, each accounting for 3.23% of the cases. Notably, BRAF mutations were observed in patients with high smoking exposure (70 pack-years), although this result may be skewed by a small sample size.
- ALK mutations were present in 9.68% of the cases, with an average residency of 8.58 years in the United States. and an average of 22.67 pack-years of smoking exposure.

These results were juxtaposed with data from Middle Eastern populations, demonstrating a greater mutation diversity among Middle Eastern Americans. 10 11 12 13 Specifically, the study found that while smoking exposure appeared to correlate with mutations in genes like KRAS and BRAF, the lower smoking rates among Middle Eastern Americans with EGFR mutations indicate that other factors may play a role in shaping mutation profiles.

## Discussion

The findings of this study underscore the significant role of smoking history in shaping the genetic mutations observed in lung carcinoma among Middle Eastern Americans. Smoking appears to be a contributing factor to the development of mutations, especially in genes like KRAS and BRAF. The association between KRAS mutations and smoking history in our study population (with an average of 27.3 pack-years) is consistent with previous studies, 14 15 16 where KRAS mutations have been linked to carcinogens present in tobacco smoke. The significant frequency of KRAS mutations (38.71%) among our study population reflects the high exposure to smoking within this group.

A consensus statement examining lung cancer patients across the Middle East and Africa (MENA) region found that 77.5% of men and 59.3% of women with lung cancer had prior smoking exposure. 17 In comparison, our study of Middle Eastern Americans observed that 76.9% of female lung cancer patients (10 out of 13) were smokers—closely mirroring, and even approaching, the regional female average. 18 19 20 On the other hand, the 61.8% smoking prevalence among male patients in our Middle Eastern American cohort (21 out of 34) is notably lower than the regional average for men (77.5%). Due to the small sample size, we know that the margin of error is larger than we would have ideally liked. Additional data would further support our claims and allow a fairer comparison between the prevalence in the MENA region and in Middle Eastern Americans. Although it is a small sample size, it is interesting to note that the prevalence of smokers among male lung cancer patients is higher in the Middle East region, while it is higher for female lung cancer patients in the Middle Eastern American population. We theorize this may be due to cultural norms and practices.

A notable finding in our study was the relatively low smoking exposure among individuals with EGFR mutations (1.25 pack-years). 9 This plausibly suggests that genetic and environmental factors, apart from smoking, might contribute to the presence of EGFR mutations. EGFR mutations are commonly found in nonsmokers or those with limited smoking history, particularly in Asian populations, and are often associated with a different clinical response to targeted therapies. The observation that EGFR mutations are present in Middle Eastern Americans, despite their relatively lower smoking exposure, suggests that this population may have a unique genetic profile, with a predominate observation of environmental, dietary, and genetic factors, similar to the same observation in Asian population.

Another intriguing finding is the rarity of BRAF mutations in the Middle Eastern American population (3.23%). While this is consistent with the low frequency of BRAF mutations in general lung cancer populations, we observed that the single BRAF mutation case had a very high smoking exposure (70 pack-years). This could suggest a potential link between intense smoking exposure and the development of BRAF mutations, but further studies with larger sample sizes are needed to confirm this association.

The lower frequency of MET mutations (3.23%) is also consistent with the general mutation profiles seen in other populations. MET mutations, though recognized as a driver in certain lung cancers, are less common than mutations in EGFR or KRAS, which may explain their rarity in this study. The relationship between MET mutations and lifestyle factors such as diet or environmental exposures, apart from smoking, remains unclear, and further research is needed to explore these potential connections.

This study provides a descriptive overview of lung cancer driver mutations among Middle Eastern Americans and compares these findings with published data from Middle Eastern populations. The term Middle Eastern is general and does not account for differences in ancestry composition and variant-calling methodologies across data sources may introduce bias. To mitigate this, analyses were limited to well-established variants, and conclusions were formed conservatively. While limited by sample size, the results highlight heterogeneity in mutation profiles and underscore the importance of further investigation in this understudied population. Consistent with prior literature, KRAS mutations were the most frequently observed and occurred predominantly among individuals with higher smoking exposure. Tobacco-related carcinogens are well known to induce KRAS mutations, and the mutation frequency observed in this cohort aligns with reports from both Western and Middle Eastern populations. However, due to the small sample size and lack of inferential testing, these observations should be interpreted as descriptive rather than confirmatory. A notable finding was the presence of EGFR mutations among individuals with minimal smoking exposure. This pattern mirrors observations in other populations, particularly among never-smokers and individuals of East Asian ancestry. While this study did not assess genetic ancestry, epigenetic markers, or environmental exposures beyond smoking, the findings raise important questions regarding the potential role of inherited susceptibility or non-tobacco-related factors in EGFR-driven lung cancer among Middle Eastern Americans. BRAF and MET mutations were rare in this cohort. The observation of a BRAF mutation in an individual with heavy smoking exposure should be interpreted cautiously, as it reflects a single case and does not support a causal association. Larger cohorts are necessary to explore whether such patterns persist. Comparisons with Middle Eastern datasets should also be interpreted with caution. The use of secondary data sources introduces potential variability related to sequencing platforms, variant-calling pipelines, and patient demographics. While these comparisons provide useful context, they do not represent direct population-level equivalence. Importantly, although lifestyle factors such as diet, physical activity, and environmental exposures are frequently discussed in the context of migration and Westernization, these variables were not directly measured in this study. Smoking remains the only lifestyle factor systematically captured, and conclusions regarding broader lifestyle influences are therefore limited. Overall, this study should be viewed as hypothesis-generating. It highlights the need for larger, multicenter studies incorporating standardized sequencing methods, detailed lifestyle data, and genetic ancestry analysis to better understand lung cancer biology in Middle Eastern American populations. No inferential statistical testing was performed due to sample size limitations.

Future studies with larger patient populations are being conducted and analyzed to include potential genetic ancestry and epigenetic mechanisms. 21

## Conclusion

This study underscores the critical role of smoking history in shaping genetic mutations in lung carcinoma within the Middle Eastern American community. Understanding these dynamics is essential for developing public health strategies that address the unique challenges faced by this population. It also highlights the need for more targeted studies that explore the intersection of genetics, smoking history, and environmental exposures. Future research should focus on expanding the scope of this investigation to better understand the implications of these findings on cancer prevention and treatment within the Middle Eastern American population.

**Table 1:** Gene mutation in our sample of Middle Eastern population living in the US compared with studies on patients with lung carcinoma in the Middle East

Gene mutation		%	Avg. years in the United States	Avg. pack-years	My Cancer Genome freq.	AUBMC freq.	EGFR freq. (Middle East and Africa)
KRAS	12	38.71%	10.42	27.3	29.61%	35.30%	–
EGFR	6	19.35%	7	1.25	22.89%	22.40%	21.20%
MET	1	3.23%	6	0	5.01%	12.90%	–
BRAF	1	3.23%	16	70	5.15%	8.20%	–
ALK	3	9.68%	13	5.83	5.44%	7.10%	–
**Total**	31 ^a^		**8.58**	**22.67**			

Abbreviations: AUBMC, American University of Beirut Medical Center; EGFR, estimated glomerular filtration rate.

A total of 31 Middle Eastern Americans with lung carcinoma underwent NGS testing; however, 1 patient had no identified mutations.

**Table 2:** Breakdown of the mutation variants in our sample of Middle Eastern population living in the United States compared with studies on patients with lung carcinoma in the Middle East

Gene mutation	Mutation variant	*N*	%	Avg. years in the United States	Avg. pack-years	My Cancer Genome freq.	AUBMC freq.	EGFR freq. (Middle East and Africa)
KRAS		12 a	38.71%	10.42	27.3	29.61%	35.30%	–
	G12C	4	12.90%					
	G12S	1	3.23%					
	G12V	3	9.68%					
	G12D	1	3.23%					
	G12A	1	3.23%					
	K117N	1	3.23%					
EGFR		6 b	19.00%	7	1.25	22.89%	22.40%	21.20%
	p.L858R	3	9.68%					
	p.G746_A750del	1	3.23%					
	p.G719S	1	3.23%					
	p.L861Q	1	3.23%					
	T790M AND EXON 19 DELETION	1	3.23%					
MET	–	1	3.23%	6	0	5.01%	12.90%	–
BRAF	–	1	3.23%	16	70	5.15%	8.20%	–
ALK	–	3	9.68%	13	5.83	5.44%	7.10%	–
**Total**		** 31 c**		**8.58**	**22.67**			

a Includes one insignificant KRAS mutation.

b A total of 6 patients had EGFR mutations; however, 1 patient had two mutation variants.

c A total of 31 Middle Eastern Americans with lung carcinoma underwent NGS testing; however, 8 patient had no significant mutations.
